# Association between Itch and Cancer in 3836 Pediatric Pruritus Patients at a Tertiary Care Center

**DOI:** 10.3390/medicines6040099

**Published:** 2019-10-05

**Authors:** Micah Belzberg, Valerie A. Larson, Raveena Khanna, Kyle A. Williams, Yevgeniy Semenov, Sonja Ständer, Anna L. Grossberg, Shawn G. Kwatra

**Affiliations:** 1 Department of Dermatology, Johns Hopkins University School of Medicine, Baltimore, MD 21231, USA; mbelzbe@jhu.edu (M.B.); vgordon4@jhmi.edu (V.A.L.); rkhanna8@jhmi.edu (R.K.); kwill184@health.fau.edu (K.A.W.); agrossb2@jhmi.edu (A.L.G.); 2 Dermatology, Washington University School of Medicine, St. Louis, MO 63110, USA; yevgeniy.semenov1@gmail.com; 3 Center for Chronic Pruritus, Department of Dermatology, University Hospital Münster, 48149 Münster, Germany; Sonja.Staender@ukmuenster.de; 4 Bloomberg School of Public Health, Johns Hopkins Bloomberg School of Public Health, Baltimore, MD 21205, USA

**Keywords:** itch, pruritus, pediatric, children, malignancy, cancer, neoplasm, epidemiology

## Abstract

**Background**: Pruritus is a well-recognized paraneoplastic phenomenon. Previous studies have examined the association of itch with a variety of malignancies in adults. However, no large study has examined this association in a pediatric population. **Methods**: A retrospective study was conducted of patients age 18 or less seen at Johns Hopkins Health System between 2012 and 2019. **Results**: A pediatric hospital population of 1,042,976 patients was reviewed. Pruritus was observed in 3836 pediatric patients of whom 130 also had cancer. Pediatric patients with pruritus were significantly more likely to have concomitant malignancy compared to pediatric patients without pruritus (OR 12.84; 95% CI 10.73–15.35, *p* < 0.001). Malignancies most strongly associated with pruritus included neoplasms of the blood (OR 14.38; 95% CI 11.30–18.29, *p* < 0.001), bone (OR 29.02, 95% CI 18.28–46.06, *p* < 0.001) and skin (OR 22.76, 95% CI 9.14–56.72, *p* < 0.001. **Conclusions**: Pruritus is significantly associated with malignancy in the pediatric hospital population. Clinicians should also be aware of the high burden of itch in pediatric malignancies and the variation in pruritus across malignancies.

## 1. Introduction

Pruritus is a well-recognized phenomenon in cancer [1]. Previous studies have examined the association of itch or pruritic dermatosis with a variety of malignancies in the adult population [2,3,4,5]. However, the pediatric population experiences significantly different ratios of malignancy types, such as higher fractions of hematologic and nervous system related cancers [6,7]. We therefore expect the association of pruritus and malignancy to be different in the pediatric population.

Sporadic case reports have presented pediatric patients with pruritus who upon investigation were subsequently diagnosed with malignancies [8,9,10,11,12]. However, to our knowledge, no study has examined the association of itch and cancer or the types of underlying cancers associated with itch in a pediatric population. The objective of this study was therefore to examine the association of pruritus and malignancy in pediatric patients at a USA tertiary care center.

## 2. Materials and Methods

A retrospective study was conducted of pediatric patients seen at Johns Hopkins Health System (JHHS), a U.S. tertiary care center which draws a diverse range of local, regional, national, and international patients. The study population consisted of all patients age 18 or less seen at JHHS between 1 January 2012 and 1 January 2019. Anonymized aggregate-level data was collected using the Slicer Dicer function within Johns Hopkins EPIC electronic medical records [3,4,5,13,14]. IRB approval was therefore waived. SNOMED-CT concept terms were used to specify all data [3,4,5,13,14]. All results combined male and female patients.

### 2.1. Pediatric Pruritus Population

Using an EPIC Slicer Dicer, 3809 pediatric patients with a visit diagnosis, billing diagnosis, or active problem list entry of “itching” were identified [3]. The comparison group was 1,039,167 pediatric patients without pruritus seen at JHHS over the same period. Each case of pruritus was also subdivided into patients with or without a skin eruption (“eruption of skin”).

### 2.2. Pediatric Cancer Population

An EPIC Slicer Dicer was used to identify all pediatric patients with a diagnosed primary malignant neoplasm (PMN). Malignancies were grouped by organ or organ system. Hematologic malignancies were further delineated using disease specific diagnoses. Separately, patients were identified in whom pruritus was diagnosed within the six months preceding their diagnosis of malignancy.

### 2.3. Statistical Analyses

Analyses compared instances of malignancy in pediatric patients with or without pruritus. Additional analyses examined instances of pruritus in each malignancy. Sub analyses were performed examining racial variation, age distribution, and the presence of concurrent kidney disease, liver disease, hyperbilirubinemia, endocrine disorders and antineoplastic medication treatment. Microsoft Excel (Redmond, WA, USA) was used to calculate odds ratios and 95% confidence intervals. Chi-squared statistics with one degree of freedom were used to calculate odds ratio *p*-values. Z-tests were used to calculate *p*-values for comparisons of odds ratios. To account for multiple comparisons, a Bonferroni correction was applied to the alpha level for each analysis (alpha = 0.05/[number of independent comparisons]).

## 3. Results

**Figure 1:**
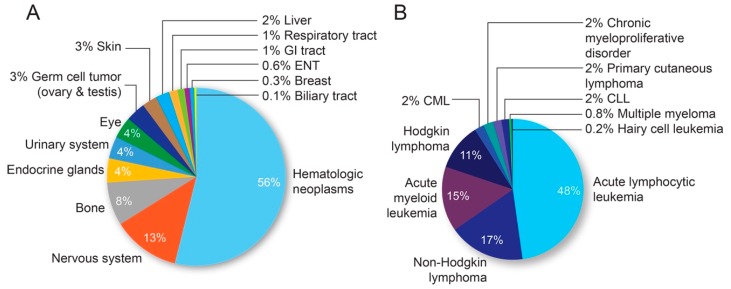
Distribution of malignancies in the pediatric population at JHHS. (**A**) Frequency of each malignancy subtype among patients age 18 or less. (**B**) Breakdown of blood malignancies as a percentage of total hematologic cancers. Chronic myeloid leukemia (CML) and Chronic lymphoid leukemia (CLL).

Between 2012 and 2019, a total of 1,042,976 pediatric patients were seen at JHHS. Demographic data are presented in Table 1. The distribution of primary malignancies identified in the pediatric hospital population is summarized in Figure 1.

### 3.1. Pruritus Is Strongly Associated with Malignancy in the Pediatric Population

**Figure 2:**
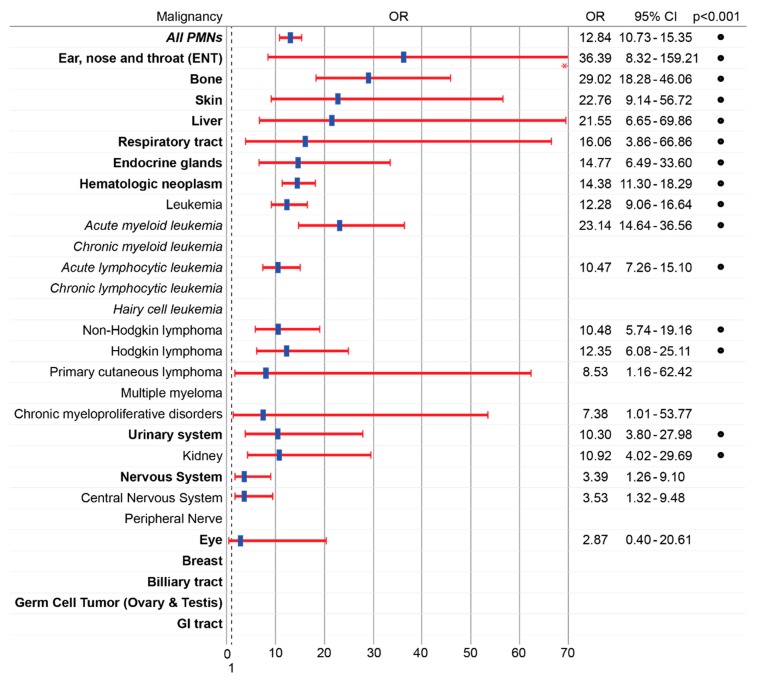
Itching is strongly associated with pediatric malignancies. Odds ratio (OR) with 95% confidence interval (95% CI) of malignancy in patients with compared to without itching in the general pediatric hospital population. Blank rows denote malignancies for which there were no concurrent diagnoses of pruritus. * 95% CI upper limit for ENT (159.2) has been truncated. • = *p* < 0.001, chi-squared test. Primary malignant neoplasm (PMN).

Between 2019 and 2012, 3809 pediatric patients with itching were treated at JHHS of whom 130 were diagnosed with a concomitant malignancy. Pediatric patients with itching were significantly more likely than pediatric patients without a diagnosis of itching to have malignancy (OR 12.84; 95% CI 10.73–15.35, *p* < 0.001) (Figure 2). PMNs strongly associated with pruritus included neoplasms of bone (OR 29.02; 95% CI 18.28–46.46, *p* < 0.001), skin (OR 22.76, 95% CI 9.14–13.63, *p* < 0.001), liver (OR 21.55; 95% CI 6.65–14.9, *p* < 0.001), and blood (OR 14.38; 95% CI 11.30–18.29, *p* < 0.001). Among hematologic dyscrasias, diagnoses most strongly associated with pruritus included acute myeloid leukemia (OR 23.14; 95% CI 14.64–36.56, *p* < 0.001), Hodgkin lymphoma (OR 12.35, 95% CI 6.08–25.11 *p* < 0.001), non–Hodgkin lymphoma (OR 10.48; 95% CI 5.74–19.16, *p* < 0.001), and acute lymphocytic leukemia (OR 10.47; 95% CI 7.26–15.10, *p* < 0.001). The weakest association was observed for neoplasms of the nervous system (OR 1.26; 95% CI 2.13 5.70, *p* = 0.009), and eye (OR 2.87; 95% CI 0.40–20.61, *p* = 0.27). No pruritus was observed in PMNs of the germ cells or biliary tract.

### 3.2. Racial Variation

Racial variation was observed in the association of pruritus and malignancy; however, upon further analyses, this variation appeared driven by racial differences in malignancy rates. Compared to Whites/Caucasians, Blacks/African Americans had significantly lower prevalence of certain neoplasms including bone, endocrine, and blood cancers. Rates of pruritus within individual malignancies were not significantly different between races.

### 3.3. The Prevalence of Skin Eruptions Varies with Underlying Malignancy

**Figure 3:**
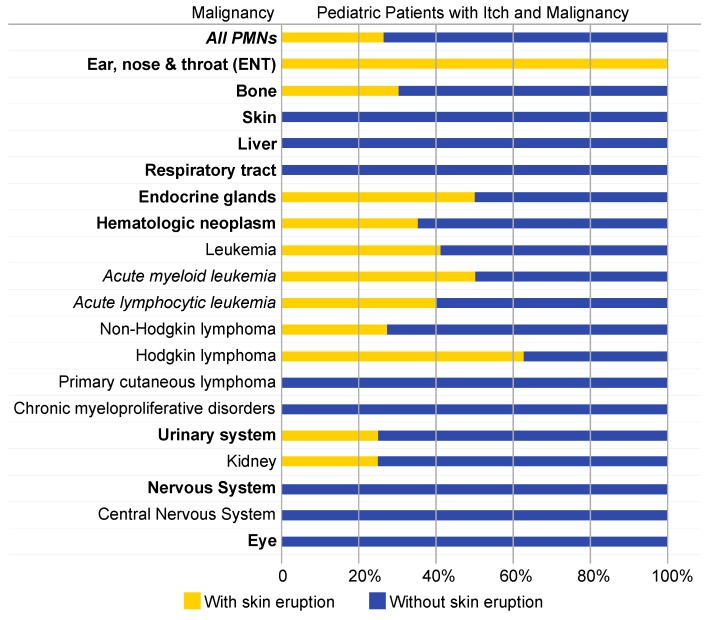
Prevalence of skin eruption in pediatric patients with itch and malignancy is variable. Lighter bars denote the percentage of patients with pruritus and a primary malignant neoplasm (PMN) who had a concurrent skin eruption. Darker bars denote percentage of patients with pruritus and a PMN without concurrent skin eruption.

Of the 3836 pediatric patients diagnosed with itching, 767 (20%) were also diagnosed with an eruption of skin. Among the 130 pediatric patients diagnosed with pruritus and malignancy, a skin eruption was observed in 34 instances (26%). The highest prevalence of skin eruption amongst pediatric patients with pruritus and malignancy were observed in PMNs of ear, nose, and throat (ENT) (100%), endocrine glands (50%), blood (35%), and bone (30%) (Figure 3). Amongst blood neoplasms, the highest prevalence was observed in Hodgkin lymphoma (63%) and leukemia (40%). No incidences of skin eruption concurrent to pruritus were observed with PMNs of the liver, nervous system, or skin. Odds ratio comparison revealed the prevalence of itching with skin eruption was significantly higher among hematologic cancers when compared to non-hematologic malignancies (OR 3.02, 95% CI 1.28–7.14, *p* < 0.01).

### 3.4. Pruritus Preceding Malignancy Is Associated with Hematologic Malignancy

Out of the total study population, 17 cases were identified in which a diagnosis of pruritus was made in the six months preceding a diagnosis of malignancy. Itch most often preadded PMNs of blood (7/17, 41%) and the gastrointestinal tract (2/17, 12%). The most frequently-diagnosed hematologic neoplasms were leukemia (3/17, 18%) and non-Hodgkin lymphoma (3/17, 18%).

### 3.5. Pruritus Among Pediatric Cancer Patients Is Associated with Comorbid Kidney Disease, Liver Disease, and Antineoplastic Use

**Figure 4:**
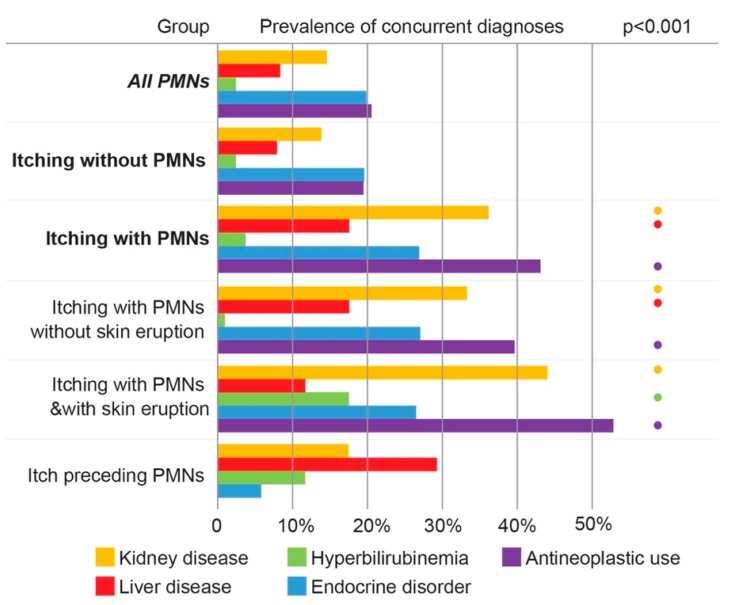
Pruritus among pediatric cancer patients is associated with comorbidities. Percentages of each group with concurrent diagnoses of kidney disease (gold), liver disease (red), hyperbilirubinemia (green), antineoplastic use (purple), and endocrine disorders (blue). • denotes statistically significant difference in comorbidity prevalence compared to pediatric patients with a primary malignant neoplasm (PMN) but without itch (*p* < 0.01).

Analyses of pediatric patients with malignancy and itch found a statistically significant (*p* < 0.01) increased incidence of concurrent kidney disease, liver disease, and antineoplastic medication use when compared to pediatric patients with malignancy but without itch (Figure 4). The subset of pediatric patients with PMNs and itching accompanied by skin eruption showed a statistically significant (*p* < 0.01) increased prevalence of hyperbilirubinemia, kidney disease, and antineoplastic use when compared with pediatric patients with cancer but without pruritus. The prevalence of these comorbidities in patients with pruritus within six months prior to their PMN diagnoses did not reach statistical significance.

## 4. Discussion

Pruritus is known to be associated with malignancies in adults, however limited data is available in the pediatric population [1,2]. While there have been sporadic pediatric cases of itch associated with diagnosed neoplasms, no large study has examined this association within a pediatric population [8,9,10,11,12]. Our findings demonstrate associations between pruritus and malignancy in a diverse pediatric population at a USA tertiary care center.

Compared to pediatric patients without itch, pediatric patients with itch were 13 times more likely to also be diagnosed with a malignancy (OR 12.84; 95% CI 10.73–15.35). By contrast, in a previous study examining the association of itch and malignancy in the adult population, patients 18 years or older with pruritus were approximately six times more likely to have concurrent malignancy as compared to adult patients without pruritus (OR 5.76; 95% CI 5.53–6.00) [3].

In the present study, the association of pruritus and malignancy was strongest for PMNs of bone, skin, liver, and blood, in particular, leukemia, non-Hodgkin and Hodgkin lymphoma. The association was weakest amongst PMNs of the eye and nervous system. These findings somewhat differ from those observed in adult patients with cancer. In the adult population, strong associations between itch and malignancy were observed in PMNs of liver, blood, and skin with weaker associations in PMNs of bone [3].

Previous publications have presented cases of pediatric patients with unexplained itch who upon investigation were subsequently diagnosed with neoplasms such as Hodgkin lymphoma, Non-Hodgkin lymphoma, T-cell lymphoma, and hepatocellular carcinoma [8,9,10,11,12]. In adults, pruritus has been observed to most frequently proceed a hematologic malignancy or occasionally gastric cancers [15,16]. This study identified 17 pediatric patients with pruritus preceding their diagnoses of malignancy. Most often these pediatric pruritic patients were later diagnosed with hematologic neoplasms (7/17, 41%). Two instances of pruritus preceding gastric malignancy were also observed. These findings suggest pediatric patients with itch and possible malignancy receive appropriate work up for hematologic neoplasms.

Subgroup analyses stratified by race revealed pronounced variation in the association of itch and malignancy. However, further analyses revealed rates of pruritus in categorical malignancies were not significantly different between races. The observed variation in association is therefore most likely due to racial differences in malignancy rates and not due to racial differences in pruritus.

The pathophysiology of pruritus in malignancy remains poorly understood [15,17]. Multiple etiologies have been described including systemic inflammation, local mass effect, antineoplastic medication use, and disruption of the hepatic or renal systems [15,18]. Hepatobiliary involvement may induce itch via accumulation of bile salts, bile acids, and bilirubin or perhaps through increased opiodergic tone [15,18,19]. Kidney invasion, ureteric obstruction, or renal failure meanwhile may induce uremic itch via the accumulation of pruritogenic metabolites or possibly a cytokine imbalance [15]. With respect to the pathogenesis of pruritus in hematologic malignancies, several mechanisms have been proposed. Previous studies have pointed to a possible role of opioid receptors owing to the observed efficacy of butorphanol in reducing pruritus in a patient with non-Hodgkin lymphoma [15,20]. Alternatively, variations in cytokine expression, known to be increased in atopic dermatitis, have also been observed in patients with both Hodgkin and non-Hodgkin lymphomas [15,21,22].

Compared to pediatric patients with malignancy but without itch, pediatric patients with PMNs and pruritus were observed to have significantly higher prevalence of kidney disease, liver disease, and antineoplastic medication use. Alternatively, patients with malignancy and itching with skin eruption showed significantly increased prevalence of hyperbilirubinemia, kidney disease, and antineoplastic use. These findings align with previously proposed mechanisms of pruritus in malignancy [15,17,18]. Additional analyses are needed to determine if these comorbidities developed as a direct result of a primary neoplastic process or secondary to antineoplastic use. Furthermore, additional studies are needed to examine the specific qualities of pruritis in malignancies and compare variation in itch intensity, location, and duration between different malignancy types.

Significant differences in pruritus rates were observed between malignancies of different systems as well as between individual malignancy types. The reason for this variation is likely multifactorial including differences in therapeutic management and effect on hepatic or renal processes [15,17,18]. With respect to hematologic malignancies, this observed variation may also be driven by differences in cytokine expression [21,22,23,24]. Additionally, very few incidences of certain cancers including hairy cell leukemia, multiple myeloma, and breast cancer were observed in our pediatric population which may limit these findings.

Data were collected through a retrospective design therefore the cause–effect relationship of these findings is limited. The tertiary care center population studied may limit the generalizability of these findings to different populations. Additional unknown confounders such as differences in socioeconomic level or other medical comorbidities could further limit these results.

## 5. Conclusions

In summary, our results indicate that pruritus is strongly associated with malignancy in the pediatric population. Malignancies strongly associated included PMNs of bone, skin, liver, endocrine glands, and blood. Additionally, several instances of itch preceding malignancy were identified. To improve patient management and diagnosis, clinicians should recognize the high burden of itch in pediatric malignancies and the variation in pruritus association across different malignancies.

## Figures and Tables

**Table 1:** Patient demographics.

Characteristic	All (*n* = 1,042,976) n (%)	Itching (*n* = 3836) n (%)	All PMNs (*n* = 2980) n (%)	PMNs + Itching (*n* = 130) n (%)
**Sex**				
Male	542,125 (52)	1542 (40)	1571 (53)	65 (50)
Female	504,788 (48)	2302 (60)	1409 (47)	65 (50)
**Race**				
White/Caucasian	515,696 (49)	1348 (35)	1539 (52)	73 (56)
Black/African American	263,166 (25)	1771 (46)	567 (19)	27 (20)
Asian	51,847 (5)	202(5)	145 (5)	10 (8)
**Age Range**				
0–6	292,033 (28)	1151(30)	805 (27)	42 (32)
6–12	365,042 (35)	1458 (38)	1013(34)	43 (33)
12–18	385,901 (37)	1228 (32)	1162 (39)	45 (35)

Sex, race, and age distribution of the study population and study subgroups. Primary malignant neoplasm (PMN).

## References

[1] Rowe B., Yosipovitch G. (2016). Malignancy-associated pruritus. Eur. J. Pain.

[2] Kilic A., Gul U., Soylu S. (2007). Skin findings in internal malignant diseases. Int. J. Dermatol..

[3] Larson V.A., Tang O., Stander S., Kang S., Kwatra S.G. (2019). Association between itch and cancer in 16,925 patients with pruritus: Experience at a tertiary care center. J. Am. Acad. Dermatol..

[4] Larson V.A., Tang O., Stander S., Miller L.S., Kang S., Kwatra S.G. (2019). Association between prurigo nodularis and malignancy in middle-aged adults. J. Am. Acad. Dermatol..

[5] Huang A.H., Canner J.K., Khanna R., Kang S., Kwatra S.G. (2019). Real-world prevalence of prurigo nodularis and burden of associated diseases. J. Investig. Dermatol..

[6] Jemal A., Siegel R., Ward E., Hao Y., Xu J., Thun M.J. (2019). Cancer statistics, 2019. CA Cancer J. Clin..

[7] Steliarova-Foucher E., Colombet M., Ries L.A., Moreno F., Dolya A., Bray F., Hesseling P., Shin H.Y., Stiller S.A. (2017). International incidence of childhood cancer, 2001-10: A population-based registry study. Lancet Oncol..

[8] De la Bretèque Amy M., Bilan P., Galesowski A., Chevallier B., Drouot D., Sigal M.L., Mahé E. (2014). Two cases of severe pruritus revealing Hodgkin’s disease in children. Annales de Dermatologie et de Venereologie.

[9] Vècsei A., Attarbaschi A., Krammer U., Mann G., Gadner H. (2002). Pruritus in pediatric non-Hodgkin’s lymphoma. Leuk. Lymphoma.

[10] Hon K.L.E., Lam M.C.A., Leung T.F., Chik K.W., Leung A.K. (2006). A malignant itch. J. Natl. Med. Assoc..

[11] Vilarinho S., Erson-Omay E.Z., Harmanci A.S., Morotti R., Carrion-Grant G., Baranoski J., Knisely A.S., Ekong U., Emre S., Yasuno K. (2014). Paediatric hepatocellular carcinoma due to somatic CTNNB1 and NFE2L2 mutations in the setting of inherited bi-allelic ABCB11 mutations. J. Hepatol..

[12] Tonnhofer U., Balassy C., Reck C.A., Koller A., Horcher E. (2009). Neuroendocrine tumor of the common hepatic duct, mimicking a choledochal cyst in a 6-year-old child. J. Pediatric. Surg..

[13] Govind K., Whang K., Khanna R., Scott A.W., Kwatra S.G. (2019). Atopic dermatitis is associated with increased prevalence of multiple ocular comorbidities. J. Allergy Clin. Immunol. Pract..

[14] Boozalis E., Tang O., Patel S., Semenov Y.R., Pereira M.P., Stander S., Kang S., Kwatra S.G. (2018). Ethnic differences and comorbidities of 909 prurigo nodularis patients. J. Am. Acad. Dermatol..

[15] Yosipovitch G. (2010). Chronic pruritus: A paraneoplastic sign. Dermatol. Ther..

[16] Zirwas M.J., Seraly M.P. (2001). Pruritus of unknown origin: A retrospective study. J. Am. Acad. Dermatol..

[17] Lidstone V., Thorns A. (2001). Pruritus in cancer patients. Cancer Treat Rev..

[18] Chiang H.C., Huang V., Cornelius L.A. (2011). Cancer and itch. Semin. Cutan. Med. Surg..

[19] Jones E.A., Bergasa N.V. (1996). Why do cholestatic patients itch?. Gut.

[20] Dawn A.G., Yosipovitch G. (2006). Butorphanol for treatment of intractable pruritus. J. Am. Acad. Dermatol..

[21] Biggar R.J., Johansen J.S., Smedby K.E., Rostgaard K., Chang E.T., Adami H.O., Glimelius B., Molin D., Dutoit S.H., Melbye M. (2008). Serum YKL-40 and interleukin 6 levels in Hodgkin lymphoma. Clin. Cancer. Res..

[22] Lee H.L., Eom H.S., Yun T., Kim H.J., Park W.S., Nam B.H., Moon-Wood S., Lee D.H., Kong S.Y. (2008). Serum and urine levels of interleukin-8 in patients with non-Hodgkin’s lymphoma. Cytokine.

[23] Petrackova M., Hamsikova E., Duskova M., Ptackova P., Klamova H., Humlova Z., Vonka V. (2016). Predictive value of serum cytokine levels in chronic myeloid leukemia patients. Immunol. Lett..

[24] Antosz H., Wojciechowska K., Sajewicz J., Choroszyńska D., Marzec-Kotarska B., Osiak M., Pajak N., Tomczak W., Baszak M.J., Baszakc J. (2015). IL-6, IL-10, c-Jun and STAT3 expression in B-CLL. Blood Cells Mol. Dis..

